# Datasets for transcriptomic analyses of maize leaves in response to Asian corn borer feeding and/or jasmonic acid

**DOI:** 10.1016/j.dib.2016.03.071

**Published:** 2016-03-28

**Authors:** Yuliang Zhang, Qixing Huang, Kayla K. Pennerman, Jiujiang Yu, Zhixin Liu, Anping Guo, Guohua Yin

**Affiliations:** a Key Laboratory of Biology and Genetic Resources of Tropical Crops, Ministry of Agriculture, Institute of Tropical Bioscience and Biotechnology, Chinese Academy of Tropical Agricultural Sciences, Haikou, Hainan 571101, China; b Department of Plant Biology and Pathology, Rutgers, The State University of New Jersey, New Brunswick, NJ 08901, USA; c Department of Agriculture, ARS, Beltsville, Agricultural Research Center, Beltsville, MD 20705, USA

**Keywords:** RNA-seq, Asian corn borer, jasmonic acid

## Abstract

Corn is one of the most widely grown crops throughout the world. However, many corn fields develop pest problems such as corn borers every year that seriously affect its yield and quality. Corn′s response to initial insect damage involves a variety of changes to the levels of defensive enzymes, toxins, and communicative volatiles. Such a dramatic change secondary metabolism necessitates the regulation of gene expression at the transcript level. In this paper, we summarized the datasets of the transcriptome of corn plants in response to corn stalk borers (*Ostrinia furnacalis*) and/or methyl jasmonate (MeJA). Altogether, 39, 636 genes were found to be differentially expressed. The sequencing data are available in the NCBI SRA database under accession number SRS965087. Our dataset will provide more scientific and valuable information for future work such as the study of the functions of important genes or proteins and develop new insect-resistant maize varieties.

**Specifications Table**

**Table:** 

Subject area	*Molecular Biology*
More specific subject area	*Volatile responses, insect herbivory, plant defense, transcriptomics*
Type of data	The *cDNA sequencing*
How data was acquired	*Illumina HiSeq 2500 sequencing platform*
Data format	*Raw reads in FASTQ format*
Experimental factors	*ACB damage, exogenous MeJA*
Experimental features	*Maize leaves were subjected to ACB feeding and/or application of exogenous MeJA before extraction of total RNA.*
Data source location	*Harbin, Heilongjiang province, China*
Data accessibility	Data is with this article are available in the NCBI SRA database under accession number SRS965087.

**Value of the data**

• The RNA-seq data allow the readers to access the transcriptomic profile of corn in response to Asian corn borer damage and MeJA stress.
• The data evaluate the utility of illumina platform for RNA-sequencing.
• Analyses of the significantly differentially expressed genes under Asian corn borer and MeJA treatment provide new targets for the development of insect-resistant maize varieties.
• Studies reveal maize defensive mechanism to the feeding of Asian corn borer and MeJA treatment.

## 1 Data

Over 50 million raw reads were generated for each experimental treatment (75,612,000 reads for maize exposed to Asian corn borer (ACB) feeding; 62,189,006 for jasmonic acid exposure; 61,528,872 for exposure to both treatments; 53,089,614 for the control). Data is available at the NCBI SRA database under accession number SRS965087.

## 2 Experimental design, materials and methods

### 2.1 Insect and plant resources

A colony of *Ostrinia furnacalis* (ACB) was maintained at College of Life Sciences, Heilongjiang University (Heilongjiang province, China) on an artificial diet (5 g vitamin C, 40 g yeast extract, 50 g barley powder, 60 g soybean powder, 14 mL ethylic acid (36%), 20 g agar powder, 1 g benzoic acid, 3 g sodium benzoate in 1000 mL ddH_2_O). The ACB were reared at 28 °C under a 14 h light: 10 h dark photoperiod. Corn seeds of variety Longdan46 were provided by the Maize Research Institute of Heilongjiang Academy of Agricultural Science and cultivated in a greenhouse at 25±1 °C, 60% humidity, and a 14 h light: 10 h dark photoperiod.

### 2.2 Insect feeding and MeJA treatments

Corn plants with four to five leaves were treated with ACB larvae (labeled as “CornOf2), MeJA (labeled as “CornJA1”), or both (labeled as “CornJAOf2”). The control group was treated with 5 mL of 0.5% ethanol/water (v:v) solution (labeled as “Corn1”). For each treatment, the entire experiment was repeated three times. Whole plants were covered with clear plastic bags. For insect feeding, twenty 1^st^ instar larvae of *Ostrinia furnacalis* (ACB) were evenly distributed on the leaves of each corn plant. The newly hatched ACB larvae from eggs are called the first instar larvae, and the 3 days old stages of 1^st^ instar larvae of similar size were chosen for each treatment. MeJA treatment involved application of 5 ml of a 225 μM MeJA solution. Control plants were treated with 5 mL of 0.5% ethanol/water (v:v) solution. The third leaf counted from the bottom of each plant was selected and immediately frozen in liquid nitrogen and stored at −80 °C for later RNA extraction.

## 3 RNA extraction

Total RNA was isolated and purified from leaf tissue using the TRIzol Plus RNA Purification Kit according to the manufacturer׳s instructions (Life Technologies). To remove any traces of genomic DNA from RNA extractions, the RNA was treated with RNase-Free DNase (Promega) as instructed by the manufacturer. Total RNA integrity and quantity were evaluated using the 2100 Bioanalyzer RNA 6000 Kit (Agilent Technologies) and the Invitrogen Qubit RNA Kit (Life Technologies). RNA samples had integrity values (RIN) between 7.6 and 9.0.

## 4 The cDNA library construction and sequencing

The TruSeq RNA Sample Prep Kit (Illumina) was used to isolate mRNA from about 5 μg of total RNA using oligo-d(T)25 magnetic beads. The mRNA was sheared with ions into ~200 nt fragments and was reverse-transcribed into cDNA. We then used the TruSeq PE Cluster Kit v3 (Illumina) to perform end repair, add an ‘A’-base to the blunt ends, and ligate the cDNA to paired-end adapters. The cDNA samples were amplified through 15 cycles of PCR. The amplification products were electrophoresed on a 2% Certified Low Range Ultra Agarose gel (Bio-Rad) and purified according to appropriate size of DNA fragments suitable for Illumina sequencing. The purified products were quantified using PicoGreen (Life Technologies) and a TBS-380 Mini-Fluorometer (Promega) and loaded on an Illumina cBot system for cluster generation by bridge PCR amplification. Sequencing was performed on an Illumina HiSeq 2500 platform.

## 5 Bioinformatic analyses

**Fig. 1:**
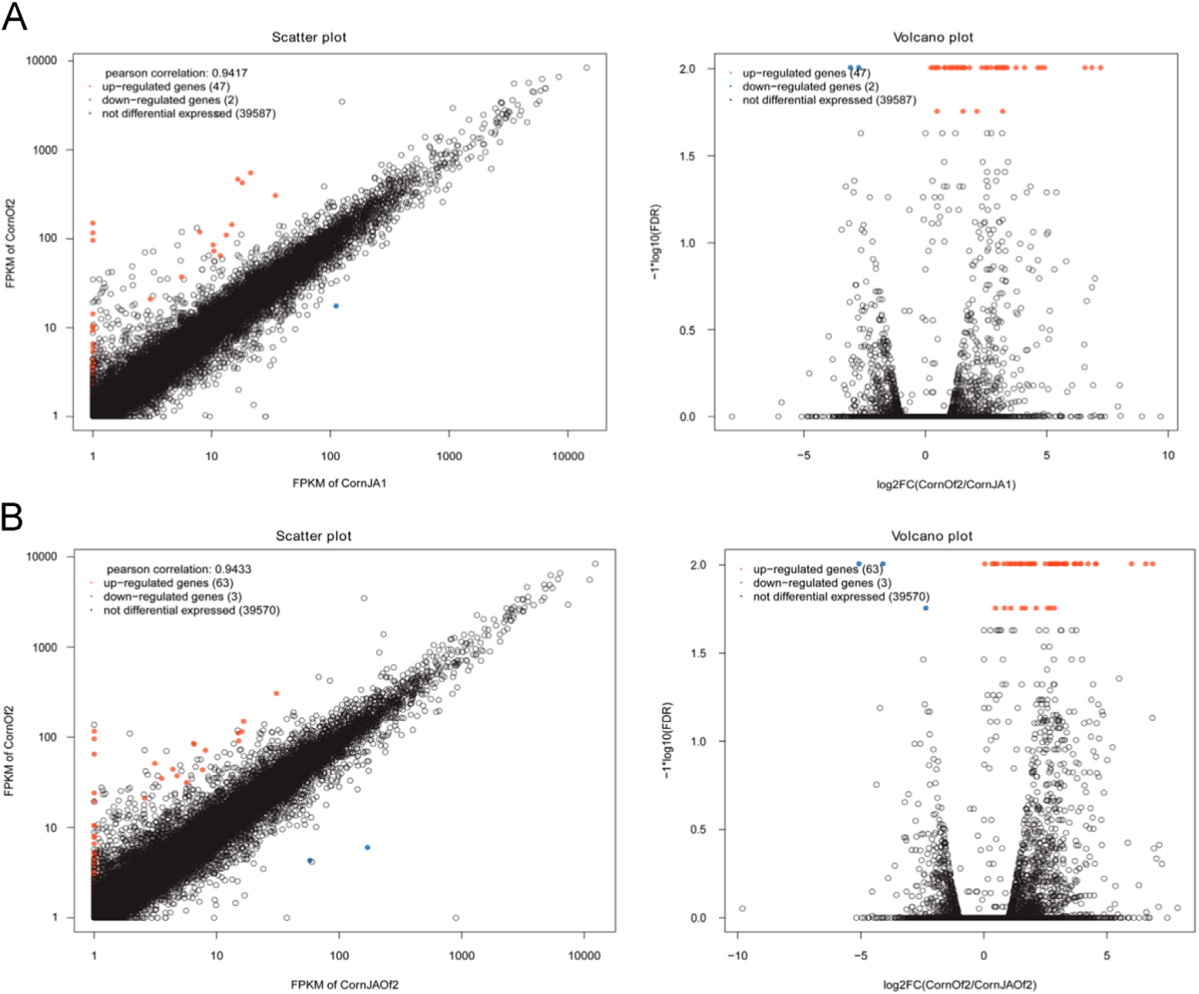
The scatter- and volcano-plots of differently expressed genes.

Standard bioinformatics analyses about this work were reported by Yang et al. [1]. We calculated gene expression values by the read/fragments per kilobase of exon per million fragments mapped (RPKM/FPKM) and the False Discovery Rate (FDR). Genes with an FPKM ratio ≥2 and FDR ≤0.05 were considered to be significantly expressed with different treatments. More strict conditions with FDR ≤0.001 and log_2_ ratio ≥2 to screen more significantly expressed genes. Altogether, 76 genes were identified to be significantly co-expressed in the different treatments using VennDiagram [2]. All these genes were listed in Supplementary Table 1. Compared between CornOf2 and CornJA1, 47 genes were up-regulated and 2 genes were down-regulated (Fig. 1A); compared between CornOf2 and CornJAOf2, 63 genes were up-regulated and 3 genes were down-regulated (Fig. 1B). New isoforms were predicted with Cufflinks (http://cufflinks.cbcb.umd.edu/) [3]. The new transcripts sequences were listed in Supplementary File 1 (fasta format), and the annotation results were in Supplementary File 2 (GTF format).

The scatter- and volcano-plots of differently expressed genes are as follows: CornOf2 vs CornJA1 (Fig. 1A) and CornOf2 vs CornJAOf2 (Fig. 1B). The left figure is the scatter-plot of differently expressed genes. The abscissa and ordinate denote the gene/transcript expression level (FPKM value) in one of two treatments. Each point represents a certain gene/transcript. The right figure is the volcano-plot of different genes. The abscissa denotes the fold changes of genes or transcripts between two treatments; the ordinate denotes the p-value.

## Footnotes

Appendix A Supplementary data associated with this article can be found in the online version at doi:10.1016/j.dib.2016.03.071.
